# Molecular insights into the HLA‐B35 molecules' classification associated with HIV control

**DOI:** 10.1111/imcb.12698

**Published:** 2023-10-09

**Authors:** Christian A Lobos, Demetra SM Chatzileontiadou, Bonin Sok, Coral‐Ann Almedia, Hanim Halim, Lloyd D'Orsogna, Stephanie Gras

**Affiliations:** ^1^ Department of Biochemistry and Chemistry, La Trobe Institute for Molecular Science La Trobe University Bundoora VIC Australia; ^2^ Department of Biochemistry and Molecular Biology Monash University Clayton VIC Australia; ^3^ Department of Clinical Immunology and PathWest Fiona Stanley Hospital Perth WA Australia; ^4^ School of Medicine University of Western Australia Perth WA Australia

**Keywords:** CD8^+^ T cell, HIV, HLA, immune response, peptide presentation

## Abstract

Human leukocyte antigen (HLA) class I molecules have been shown to influence the immune response to HIV infection and acquired immunodeficiency syndrome progression. Polymorphisms within the HLA‐B35 molecules divide the family into two groups, namely, Px and PY. The Px group is associated with deleterious effects and accelerated disease progression in HIV^+^ patients, whereas the PY group is not. The classification is based on the preferential binding of a tyrosine at the C‐terminal part of the peptide in the PY group, and a nontyrosine residue in the Px group. However, there is a lack of knowledge on the molecular differences between the two groups. Here, we have investigated three HLA‐B35 molecules, namely, HLA‐B*35:01 (PY), HLA‐B*35:03 (Px) and HLA‐B*35:05 (unclassified). We selected an HIV‐derived peptide, NY9, and demonstrated that it can trigger a polyfunctional CD8^+^ T‐cell response in HLA‐B*35:01^+^/HIV^+^ patients. We determined that in the complex with the NY9 peptide, the PY molecule was more stable than the Px molecule. We solved the crystal structures of the three HLA molecules in complex with the NY9 peptide, and structural similarities with HLA‐B*35:01 would classify the HLA‐B*35:05 within the PY group. Interestingly, we found that HLA‐B*35:05 can also bind a small molecule in its cleft, suggesting that small drugs could bind as well.

## Graphical abstract

Our work provides a deeper understanding of the classification of HLA‐B35 molecules regarding their influence on HIV infection outcomes. Stability and structural studies revealed differences among HLA‐B35 molecules, which led to the classification of HLA‐B*35:05 into the PY group. We also revealed the binding of small molecule in HLA‐B*35:05 cleft, suggesting a potential for other small molecules such as drugs to bind this HLA.

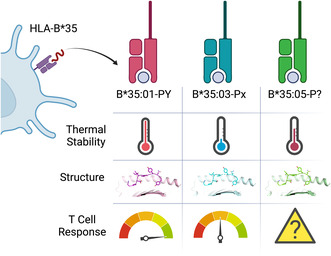

## INTRODUCTION

There have been extensive studies that have strongly associated HIV‐I infection and acquired immunodeficiency syndrome (AIDS) progression with the expression of specific human leukocyte antigen class I (HLA‐I) alleles. Studies on cohorts of long‐term nonprogressors revealed that rare individuals are able to maintain HIV viral titers to very low amounts (< 400 copies of HIV RNA/mL of plasma) with little to no need for antiretroviral therapy.^1^ The ability of long‐term nonprogressors to limit disease progression is linked to the expression of “protective” HLA‐I molecules such as HLA‐B*57:01,^2^, ^3^ HLA‐B*27:05^4^ or the more recently discovered HLA‐B*52:01,^5^ which are recognized by CD8^+^ T cells.^6^ Although the exact mechanisms remain unclear, protective alleles have a dominant role in influencing disease outcomes through influencing CD8^+^ T‐cell activation and repertoires.^4^ A strong CD8^+^ T‐cell response helps long‐term nonprogressors maintain a higher CD4^+^ T‐cell count over time and rarely progress to the development of AIDS. Investigation of these mechanisms behind the impact on HIV‐I disease provides insights into the protective immunity against HIV‐I and how it can be manipulated for therapeutic purposes.^7^

Interestingly, a study found that HIV^+^ individuals expressing HLA‐B35 have different disease outcomes depending on which allomorph they express, with some allomorphs being implicated in accelerated AIDS progression, whereas others having no impact on the disease progression, despite differing by as few as one amino acid.^8^ The HLA‐I molecules present small peptides (8–10 residues), derived from pathogens or self‐proteins, on the cell surface. The peptides are bound in the HLA antigen–binding cleft into six major pockets designated A through F.^9^ Peptides are anchored to the cleft by two main anchor residues, the second residue at position 2 (P2) and the last residue of the peptide (PΩ), that bind into the B and F pocket, respectively.^9^ The HLA‐I–binding pockets have different biochemical properties that lead to allele‐specific peptide motifs.^9^, ^10^, ^11^ HLA polymorphisms can influence the disease progression of HIV‐I infection,^12^ and are mainly localized in the B and F pockets of the HLA molecules.

Currently, the classification of the HLA‐B35 family into two groups (PY and Px), in the context of HIV‐I infection, is based on their binding preference at the F pocket to specific C‐terminal anchor residues of the peptide (PΩ). Both PY and Px share a B pocket that binds proline at P2 of the peptide. The HLA‐B35 molecules in the PY group are selective for tyrosine at PΩ, while Px molecules bind a residue other than tyrosine at PΩ.^8^ The selective difference is attributed to an amino acid polymorphism in the HLA heavy chain 116, with a small serine or a larger phenylalanine/tyrosine in the PY and Px groups, respectively, changing the size of the F pocket.^9^, ^13^ Based on these motifs, several alleles of the HLA‐B35 family have been classified into either Px or PY groups. The amino acid differences between these groups have been linked to a variable rate of disease progression, with HLA‐B35 Px exhibiting deleterious effects.^14^ As HLA‐I restricts CD8^+^ T cells,^15^ the difference in disease progression between HLA‐B35 PY and Px groups was believed to be a result of the CD8^+^ T‐cell response. There have been studies that show no discernible difference between PY and Px CD8^+^ T‐cell responses when presenting HIV‐I epitopes recognized by both groups,^16^, ^17^ despite a 3‐year faster progression to AIDS in HLA‐B35‐Px^+^ patients compared with HLA‐B35‐PY^+^ individuals.^16^ In addition, because both PY and Px HLA‐B35 can present identical HIV‐I epitopes, the T‐cell response might not be the only factor to impact the disease outcome.^18^ Indeed, a study on the major histocompatibility complex type I inhibitory molecule, immunoglobulin‐like transcript 4, has shown a higher affinity for the HLA‐B*35:03 from the Px group than for the HLA‐B*35:01 (PY group), which could in turn impact on disease progression differently in the context of HIV infection.^18^ By contrast, even though HLA‐B*35‐PY subtypes have no detectable impact on HIV‐1 disease outcomes, one study showed rapid disease progression in HIV clade B infection linked to HLA‐B*35:01 expression, specifically to a single Gag epitope (NY10), an association that does not hold in two large clade C–infected African cohorts.^19^

To provide a further understanding of the differences and the molecular mechanism between the HLA‐B35 molecules from the Px and PY groups, our study focused on determining the biophysical differences between one PY molecule (HLA‐B*35:01), one Px molecule (HLA‐B*35:03) as well as one unclassified HLA‐B35 molecule (HLA‐B*35:05), which was found to have a protective effect in HIV‐1 subtype AE infection.^20^ The HLA‐B*35:01 allele is relatively frequent in people of European (6%), Asian (5%) and African (3.4%) descent. HLA‐B*35:03 is less frequent than HLA‐B*35:01 (2.8%, 5.2%, 2.3%, respectively), and HLA‐B*35:05 is rarer in European (0.03%) than in Asian (0.86%) and African (0.77%) people (% frequencies from http://www.allelefrequencies.net/). We used the NY9 peptide (^330^NPDIVIYQY^338^) derived from HIV‐I reverse transcriptase as it is recognized by CD8^+^ T cells in HIV‐I–infected patients expressing HLA‐B35‐PY or HLA‐B35‐Px alleles.^18^ The NY9 peptide was selected as it was shown to be a dominant T‐cell epitope in both HLA‐B*35:01^+^ ^19^, ^21^ and HLA‐B*35:03^+^ HIV^+^ patients.^18^ We first confirmed that the CD8^+^ T cells can recognize NY9 in HLA‐B*35:01^+^HIV^+^ antiretroviral therapy–naïve patients, and demonstrated that the response is polyfunctional. We then determined the ability of each HLA‐B35 molecule to form a stable complex with the NY9 epitope and showed that HLA‐B*35:03 and to a lesser extent HLA‐B*35:05 molecules were less stable than HLA‐B*35:01. Structural determination of the three HLA‐B35‐NY9 complexes allowed for the comparison between the Px and PY molecules, as well as the unclassified allomorph, and revealed differences in the antigen‐binding cleft between HLA‐B35 molecules when presenting the same peptide. Overall, our data provide molecular insights into the classification of Px and PY groups, as well as further classification of the HLA‐B*35:05 into the PY group. In addition, we discovered that HLA‐B*35:05 can bind small molecules in its cleft, buried underneath the peptide, which might provide a link for the drug hypersensitivity observed in HLA‐B*35:05^+^HIV^+^ patients.

## RESULTS

### Polyfunctional T‐cell response to NY9 in HLA‐B*35:01^+^HIV^+^ patients

We previously enrolled over 300 HIV^+^ patients for our study, from whom we have defined the HLA expression profile as well as collected blood, before initiation of antiretroviral therapy, and isolated peripheral blood mononuclear cells (PBMCs).^22^, ^23^ From this cohort, 26 patients were expressing an *HLA‐B35* allele, 19 from the PY group and 7 from the Px group (Table 1). From the PY group, 5/19 (26%) had a low viral load (< 400 copies mL^−1^), whereas from the Px group, 1/7 (14%) had a low viral load. The number of patients expressing a *HLA‐B35* allele in this cohort is too small for statistical significance to be drawn, but interestingly the trend of the PY group being linked to better outcomes and protection from AIDS progression is consistent with previous published work.^8^, ^16^

**Table 1:** HIV^+^ HLA‐B35^+^ patients enrolled in our study.

Patient ID	Viral load (copies mL^−1^)	HLA‐B	Sex	Age (years)	Group
PY‐D1	400	35:01	M	63	PY
PY‐D2	400	35:01	M	64	PY
PY‐D3	400	35:01	M	47	PY
PY‐D4	56 661	35:01	M	57	PY
*PY‐D5*	50	35:01^a^	F	44	PY
PY‐D6	3631	35:01^a^	F	40	PY
PY‐D7	6310	35:01^a^	F	41	PY
PY‐D8	3548	35:01^a^	F	55	PY
PY‐D9	20 893	35:01^a^	F	88	PY
PY‐D10	169 824	35:01	M	36	PY
PY‐D11	7763	35:01	M	45	PY
PY‐D12	29 512	35:01	M	59	PY
PY‐D13	6761	35:01	F	46	PY
PY‐D14	478 630	35:01^a^	M	60	PY
PY‐D15	2344	35:01^a^	M	61	PY
PY‐D16	7588	35:01^a^	F	68	PY
PY‐D17	107 152	35:01^a^	M	50	PY
PY‐D18	83 176	35:01^a^	M	49	PY
PY‐D19	20 417	35:08	M	38	PY
Px‐D1	100 000	35:02	M	40	Px
Px‐D2	100 000	35:02	M	58	Px
Px‐D3	100 000	35:02	M	55	Px
Px‐D4	999 999	35:02	M	54	Px
Px‐D5	40	35:02	M	61	Px
Px‐D6	7943	35:03	M	77	Px
Px‐D7	131 826	35:03 (or 35:70)	M	50	Px

The sample used for TCR sequencing (PY‐D5) and T‐cell clones' generation is indicated in italics.

F, female; HLA, human leukocyte antigen; M, male.

^a^ B35:01 or rare alleles B*35:40 N/B*35:42/B*35:57/B*35:27, unresolved from the HLA typing.

**Figure 1:**
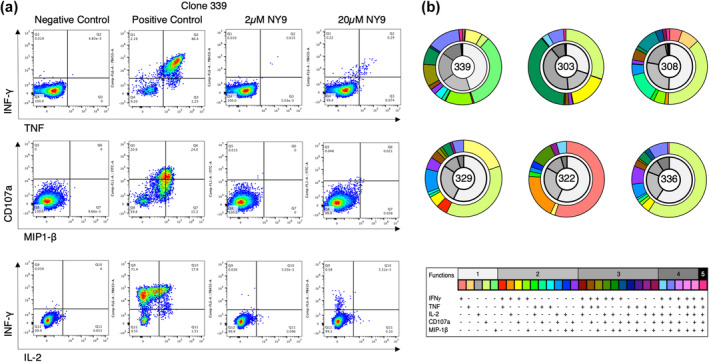
Assessment of the HLA‐B*35:01‐NY9‐induced CD8^+^ T‐cell responses. **(a)** Representative flow plots displaying 339 CD8^+^ T‐cell clone's IFN‐γ, TNF, MIP‐1β, IL‐2 and CD107a responses to the NY9 peptide at 2 or 20 μm, or the negative (no peptide) and positive (phorbol myristate acetate/ionomycin nonspecific stimulators) controls. **(b)** Pie charts displaying the polyfunctionality of the six NY9‐specific CD8^+^ T clones. The frequency of CD8^+^ T cells with different cytokine production profiles (IFN‐γ, TNF, IL‐2, MIP‐1β and CD107a) was determined, along with the number of different cytokines produced, minus the no‐peptide control. The outer ring of the double ring pie shows the cytokine profile in different colors (as per the table below) and the inner ring represents the number of cytokines produced with black for five and white for one. HLA, human leukocyte antigen; IFN, interferon; IL, interleukin; MIP, macrophage inflammatory protein; TNF, tumor necrosis factor.

We first assessed the level of response toward the NY9 epitope in HLA‐B*35:01^+^HIV^+^ patients. To test this, we isolated PBMCs from an HLA‐B*35:01^+^HIV^+^ controller individual (PY‐D5, Table 1). After staining the PBMCs with HLA‐B*35:01‐NY9 tetramer, we single‐cell sorted the tetramer‐positive CD8^+^ T cells and clonally expanded them. We used six CD8^+^ T‐cell clones to undertake intracellular cytokine staining and determine their ability to produce cytokines upon presentation of the NY9 peptide (Figure 1, Supplementary figure 1). All the clones were highly polyfunctional in response to the presentation of the NY9 epitope, with three clones expressing all the five cytokines tested (clones 339, 303 and 308), while the three others expressed up to four cytokines (clones 329, 322 and 336; Figure 1b). The data confirmed that the NY9 is a strong T‐cell epitope in HLA‐B*35:01^+^ HIV^+^–derived samples and an HIV epitope restricted to this HLA.

### HLA‐B35‐PY molecule exhibits higher stability than HLA‐B35‐Px in complex with NY9

As we confirmed that the NY9 peptide can activate CD8^+^ T cells in HIV^+^ donors carrying an HLA‐B*35:01 allele (PY group), this peptide could be used to further explore the differences between PY and Px groups. HLAs are extremely polymorphic molecules in humans, with over 25 000 HLA‐I alleles identified.^24^, ^25^ The alignment of the HLA‐B35–binding cleft sequences (amino acids 1–180, Table 2) showed that HLA‐B*35:03 (Px group, Phe/Tyr116) differs from HLA‐B*35:01 (PY group, Ser116) by one amino acid at position 116, whereas HLA‐B*35:05 differs from HLA‐B*35:01 by three amino acids at positions 94, 95 and 97 (Table 2). A previous study showed that the residues at positions 114 and 116 of the HLA cleft were critical for the ability, or lack of, to bind a PΩ‐Tyr peptide residue.^8^ The presence of an aromatic residue at position 116 (Px group) is believed to abrogate the binding of a peptide containing a PΩ‐Tyr.^8^ As HLA‐B*35:05 and HLA‐B*35:01 have conserved residues at positions 114 and 116 (Table 2), it would be anticipated that the HLA‐B*35:05 could accommodate a PΩ‐Tyr in its F pocket.

**Table 2:** Amino acid sequence alignment of HLA‐B35 molecules at peptide‐facing residues of the antigen‐binding cleft.

Classification	HLA molecule	Peptide‐binding site amino acid position							Preferred anchor residue	
		94	95	97	109	114	116	156	P2	PΩ
PY	B*35:01	I	I	R	L	D	S	L	P	Y
	B*35:08	—	—	—	—	—	—	*R*	P	Y
Px	B*35:02	—	—	—	*F*	*N*	*Y*	—	P	L/V
	B*35:03	—	—	—	—	—	*F*	—	P	L/M
Unclassified	B*35:05	*T*	*L*	*S*	—	—	—	—	P	F

Sequence alignment of amino acids that face into the antigen‐binding cleft of HLA‐B35‐PY, ‐Px and unclassified alleles. Italicized amino acids are those that deviate from the sequence of HLA‐B*35:01. The HLA‐B35 alleles are organized by classification and listed alongside preferred anchor residues (adapted from Gao *et al.* ^8^).

HLA, human leukocyte antigen.

To determine whether HLA‐B35 polymorphism could impact the NY9 presentation, we refolded each HLA‐B35 allomorph's heavy chain separately with the NY9 peptide and β2‐microglobulin (β2m).^26^ All three HLA‐B35 molecules successfully refolded,^18^ showing that the presence of Phe116 in HLA‐B*35:03 does not abrogate the binding of NY9 that contains a PΩ‐Tyr, in line with previous studies, but in contrast to others.^27^ This clearly shows that both HLA‐B35‐PY and HLA‐B35‐Px molecules can present identical HIV‐I epitopes.

**Figure 2:**
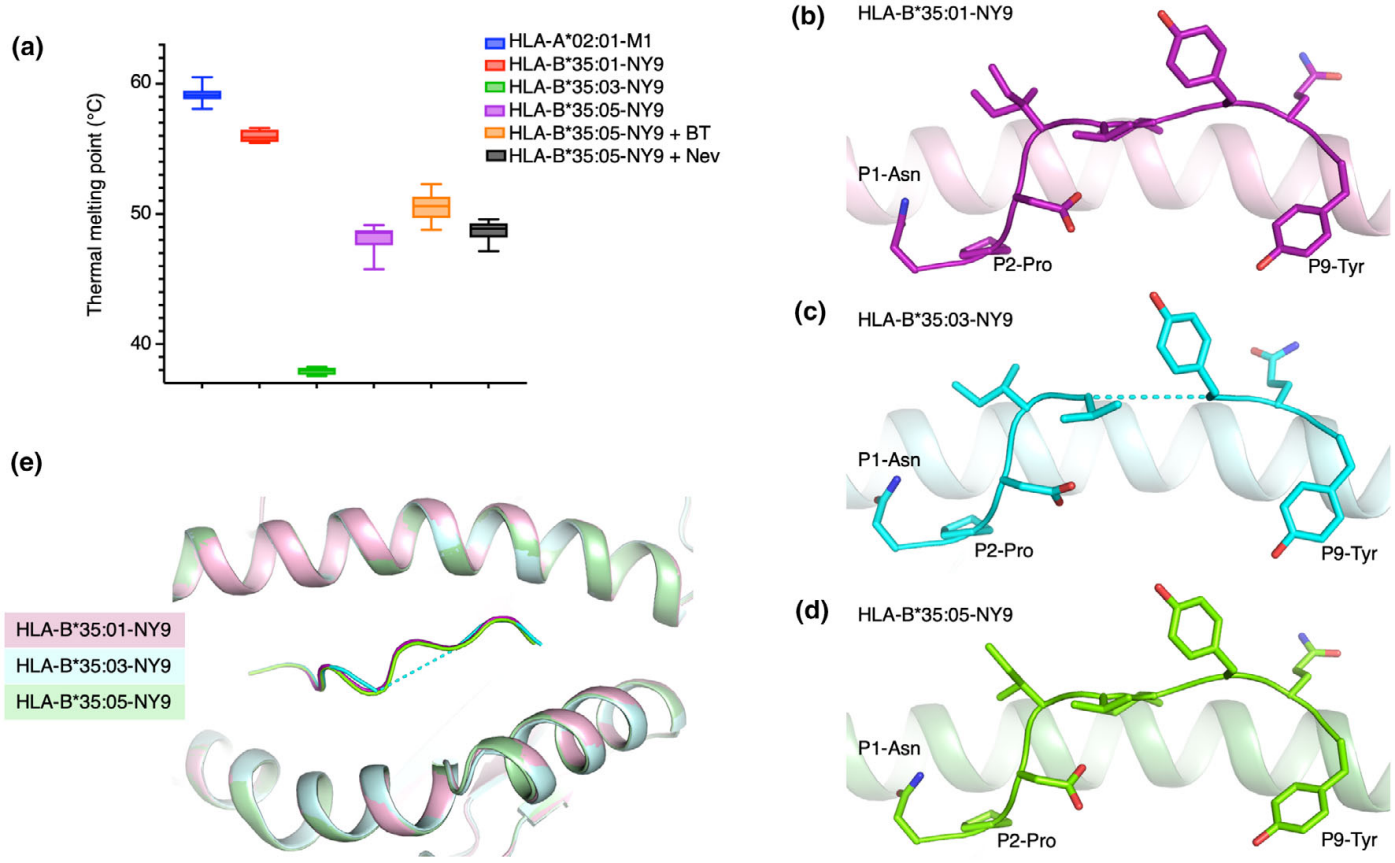
Molecular characteristic of the HLA‐B35 molecules from the Px and PY groups. **(a)** Graph displaying the thermal unfolding of HLA‐B*35:01, HLA‐B*35:03 and HLA‐B*35:05 in complex with NY9, as well as of HLA‐B*35:05‐NY9 with Bis‐Tris (BT) or nevirapine (Nev), summarized by thermal melting temperature. The HLA‐A*02:01‐M1 complex was used as a control. The data represent the mean of three independent experiments and the standard error of the mean is shown by the bar. Structures of the NY9 peptide presented by **(b)** HLA‐B*35:01 (pink), **(c)** HLA‐B*35:03 (cyan) and **(d)** HLA‐B*35:05 (green). The peptide is shown as sticks. **(e)** Superimposition of the antigen‐binding clefts (residues 1–180) of HLA‐B*35:01‐NY9, HLA‐B*35:03‐NY9 and HLA‐B*35:05‐NY9 complex structures. The peptide is shown as a ribbon. The dashed line represents the missing part of the peptide in the structure of the NY9 peptide presented by the HLA‐B*35:03 molecule.

The stability of the peptide–HLA (pHLA) complex is an important factor for peptide immunogenicity.^28^, ^29^ We therefore determined the stability of HLA‐B*35:01‐NY9, HLA‐B*35:03‐NY9 and HLA‐B*35:05‐NY9 using thermal melt assay. HLA‐B*35:01‐NY9 (PY) exhibited the highest thermal stability with a T_m_ (thermal melting temperature) of ~56°C, while the HLA‐B*35:03 (Px) exhibited a T_m_ of ~38°C (Figure 2a, Supplementary figure 2, Table 3), which is very low compared with other HLA‐B35 complexes.^30^ This result is in line with the Px group molecules definition that does not favor a PΩ‐Tyr in the peptide (Table 2), and so this is consistent with the classification of HLA‐B*35:03 within the Px group. The unclassified HLA‐B*35:05 in complex with the NY9 peptide had a T_m_ of 48°C, a value close to the one observed for HLA‐B*35:01 from the PY group.

**Table 3:** Thermal stability of the HLA‐B35‐NY9 complexes.

Complexes	T_m_ (°C)	Group
HLA‐B*35:01‐NY9	56.0 ± 0.2	‐PY
HLA‐B*35:03‐NY9	38.0 ± 0.1	‐Px
HLA‐B*35:05‐NY9	48.1 ± 0.4	Unclassified
HLA‐B*35:05‐NY9 + Bis‐Tris	50.6 ± 0.5	
HLA‐B*35:05‐NY9 + nevirapine	48.7 ± 0.4	

Thermal unfolding of the HLA‐B35‐NY9 complexes summarized by thermal melting temperature (T_m_) and standard error of the mean values.

HLA, human leukocyte antigen.

### Molecular characteristics of the HLA‐B35 molecules from the Px and PY groups

To investigate the impact of polymorphisms and the basis of the classification of HLA‐B35 allomorphs into the two groups, we solved the HLA‐B*35:01‐NY9, HLA‐B*35:03‐NY9 and HLA‐B*35:05‐NY9 structures using X‐ray crystallography at high resolution (Table 4). The electron density was clear in the antigen‐binding cleft, which allowed an in‐depth investigation of the impact of HLA polymorphisms (Supplementary figure 3).

**Table 4:** Crystallographic data collection and refinement statistics of HLA‐B35‐NY9 complexes.

	HLA‐B*35:01‐NY9	HLA‐B*35:03‐NY9	HLA‐B*35:05‐NY9
Data collection statistics			
Space group	P3	P2_1_2_1_2_1_	P2_1_2_1_2_1_
Cell dimensions (a, b, c) (Å)	157.82, 157.82, 42.39	50.90, 81.71, 110.02	50.88, 81.90, 109.32
Resolution (Å)	78.92–1.74 (1.77–1.74)	46.19–1.55 (1.94–1.90)	46.12–1.73 (1.76–1.73)
Total number of observations	209 488 (10 706)	181 802 (11 345)	402 596 (22 293)
Nb of unique observations	40 150 (2099)	36 900 (2304)	48 556 (2637)
Multiplicity	5.2 (5.1)	4.3 (4.9)	8.3 (8.5)
Data completeness (%)	99.6 (96.7)	99.9 (100.0)	100.0 (100.0)
I/σ_I_	13.4 (1.3)	7.5 (1.6)	9.2 (1.6)
*R*_pim_ ^a^ (%)	2.6 (41.8)	7.0 (48.5)	2.3 (40.7)
CC_1/2_ (%)	99.3 (69.5)	99.2 (69.6)	99.7 (77.3)
Refinement statistics			
*R*_factor_ ^b^ (%)	18.6	20.1	19.2
*R*_free_ ^b^ (%)	22.1	23.5	22.1
Root‐mean‐square deviation from ideality			
Bond lengths (Å)	0.0083	0.0083	0.0082
Bond angles (°)	0.94	0.92	0.93
Ramachandran plot (%)			
Favored/allowed	100	100	100
Disallowed	0	0	0
PDB code	7SIG	7SIH	7SIF

Values in parentheses are for the highest resolution shell.

CC_1/2_, correlation coefficient; HLA, human leukocyte antigen.

^a^ *R*_p.i.m_ = ∑_hkl_ [1/(*N*−1)]^1/2^ ∑_i_ | *I*_hkl_, _i_ −< *I*_hkl_ > | / ∑_hkl_ < *I*_hkl_>.

^b^ *R*_factor_ = ∑_hkl_ | |*F*_o_ | − | *F*_c_ | | / ∑_hkl_ | *F*_o_ | for all data except ≈ 5%, which were used for *R*_free_ calculation.

**Figure 3:**
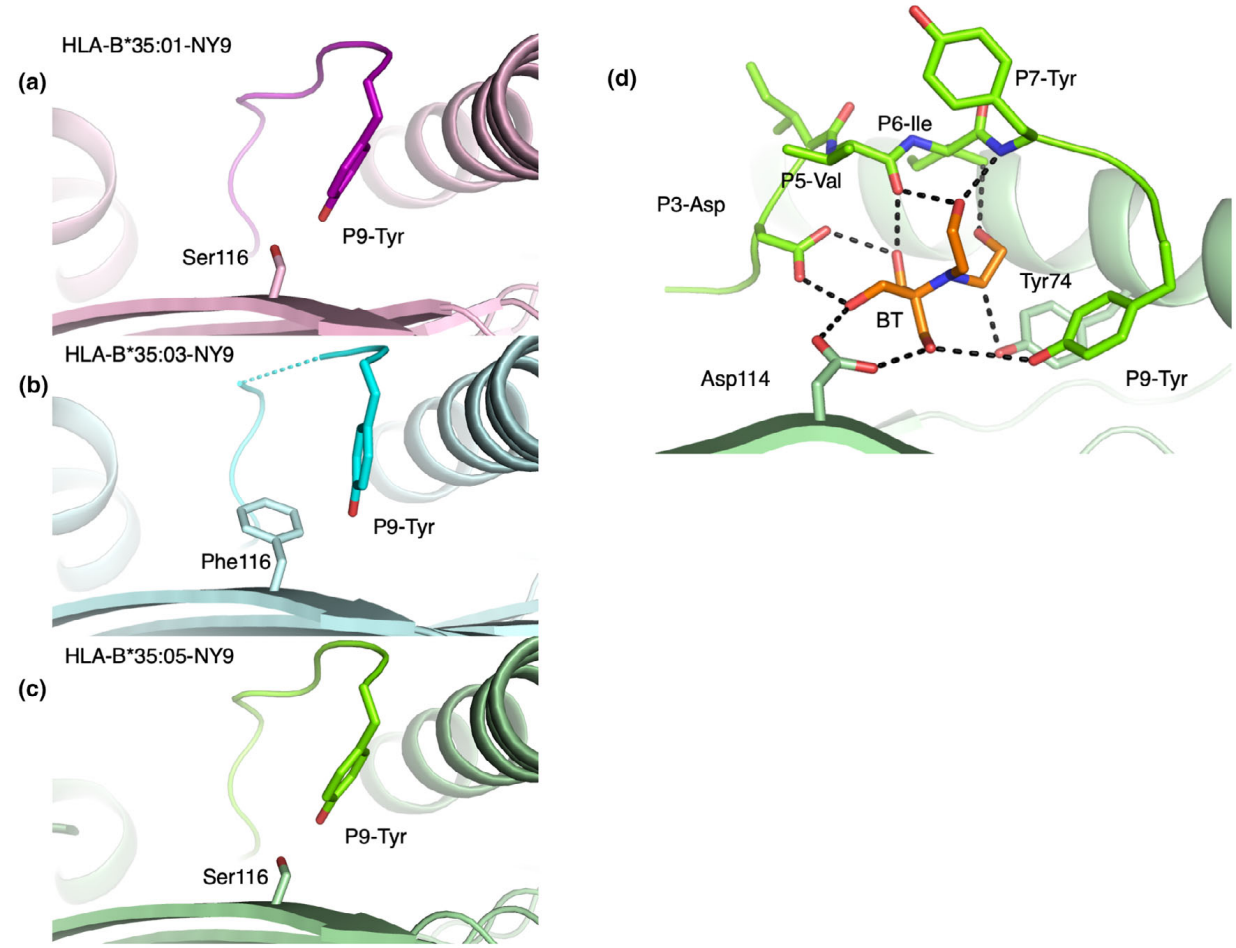
Structural analysis of the F pocket and molecular docking of the drug in the human leukocyte antigen (HLA). Side view of the cleft that shows the positioning of P9‐Tyr of the peptide and the residue 116 of the **(a)** HLA‐B*35:01‐NY9 (pink), **(b)** HLA‐B*35:03‐NY9 (cyan) and **(c)** HLA‐B*35:05‐NY9 (green). **(d)** The interaction network of the Bis‐Tris (BT) molecule is shown in orange, with the NY9 peptide (green sticks) and HLA‐B*35:05 (pale green) residues. Key interactions are represented as black dashed lines.

The HLA‐B*35:01‐NY9 structure (Figure 2b) revealed a canonical orientation of the peptide in an extended conformation, with the main anchor residues P2‐Pro and P9‐Tyr binding to the B and F pocket, respectively. The central part of the NY9 peptide adopted a flat conformation in the cleft, and there was no secondary anchor residue that was seen before for HLA‐B*35:01.^31^ We solved and compared the structures of HLA‐B*35:03‐NY9 (Figure 2c) and HLA‐B*35:05‐NY9 (Figure 2d) with HLA‐B*35:01‐NY9 (Figure 2b). Superimposition of the HLA antigen–binding clefts (residues 1–180; Figure 2e) did not reveal large differences with a root‐mean‐square deviation of ~0.20 Å. While the NY9 peptide adopted the same conformation when presented by HLA‐B*35:01 and HLA‐B*35:05 (root‐mean‐square deviation of 0.14 Å), it was different in HLA‐B*35:03 (root‐mean‐square deviation of 0.35 Å; Figure 2e). The electron density for the NY9 peptide presented by the HLA‐B*35:03 molecule was not well defined, especially its central part (Supplementary figure 3b, e). This high mobility of the central part of the NY9 peptide was attributed to the large aromatic residue Phe116, compared with Ser116 in HLA‐B*35:01, that pushed the P9‐Tyr toward the C‐terminal part of the F pocket (by 2.2 Å for the P9‐Tyr hydroxyl group, Figure 3a, b). The larger Phe116 of HLA‐B*35:03 is most likely favorable to the binding of a small residue at PΩ of the peptide (Table 2, Figure 3b) instead of a tyrosine. By contrast, the HLA‐B*35:05 allomorph has a Ser116 favoring the PΩ‐Tyr to dock in a similar position as observed in the HLA‐B*35:01 molecule (Figure 3c). This is also consistent with the higher thermal stability observed for both HLA‐B*35:01 and HLA‐B*35:05 compared with HLA‐B*35:03, altogether suggesting that the unclassified HLA‐B*35:05 allomorph belongs to the PY group.

### HLA‐B*35:05 crystallized with a small molecule located underneath the peptide in the cleft

Surprisingly, the analysis of the HLA‐B*35:05 structure revealed that the cleft was filled with a molecule of Bis‐Tris. The Bis‐Tris was used only during the crystallization of the protein. Of note, out of the 300 different crystallization solutions screened for the HLA‐B*35:05‐NY9 complex, only three of them led to the formation of crystals, all containing Bis‐Tris.

The small molecule of Bis‐Tris sits centrally in the cleft underneath the peptide interacting with P3‐Asp, P5‐Val, P6‐Ile and P7‐Tyr of the NY9 peptide, as well as with Asp114 and Tyr74 from the HLA‐B*35:05 molecule *via* a large network of hydrogen bonds and van der Walls contacts (Figure 3d). A key characteristic of the HLA‐B*35:05 cleft that differs from the two other allomorphs studied here is the presence of a small residue in the D/E pockets that creates enough room for a small molecule to fit in the central part of the antigen‐binding cleft. This was also facilitated by the lack of secondary anchor residues of the NY9 peptide to bind to the HLA‐B*35 allomorphs, which would otherwise clash with the Bis‐Tris molecule. The binding of small molecules or drugs has been reported for HLA molecules. For example, HLA‐B*57:01 can bind the drug abacavir deep in its cleft, leading to a change of self‐peptide repertoire presented by the HLA molecule.^32^ This change in the peptide repertoire leads to abnormal T‐cell activation and is believed to be responsible for abacavir drug hypersensitivity in HLA‐B*57:01^+^HIV^+^ patients. Interestingly, the HLA‐B*35:05 molecule has been linked to nevirapine drug allergies that led to skin rash in HIV^+^ patients in a cohort in Thailand.^33^, ^34^, ^35^

Therefore, we assessed whether the presence of the Bis‐Tris or nevirapine could further stabilize the HLA‐B*35:05‐NY9 complex. The Bis‐Tris was added in half of the concentration used in the crystallization condition (i.e. approximately two times in excess of the HLA heavy chain), whereas the concentration of nevirapine was six times in excess of the HLA heavy chain, comparable to a previous study with abacavir drug.^32^ The stability of the HLA‐B*35:05‐NY9 complex was moderately increased by 2.5°C in the presence of Bis‐Tris in solution (*P* = 0.0001, 95% CI −3.8 to −1.0, Tukey's honestly significant difference test), but not in the presence of nevirapine (*P* = 0.7864, 95% CI −2.03 to 0.82, Tukey's honestly significant difference test; Table 3, Figure 2a). We tried to cocrystallize the HLA‐B*35:05‐NY9 complex with nevirapine by refolding the drug with the HLA or by adding it to the crystallization condition (as per the Bis‐Tris), but, unfortunately, we were only able to obtain poor‐quality crystals that did not lead to structure determination.

## DISCUSSION

The current discourse regarding the HLA‐B35 family is that individuals who express HLA‐B35 molecules that belong to the Px group are prone to faster HIV‐1 disease progression to AIDS than individuals who belong to the HLA‐B35‐PY group.^8^ To understand the underlying molecular mechanism behind this classification, we have studied the stability, flexibility and structural features of the unclassified HLA‐B*35:05 molecule, and of HLA‐B*35:01 (PY) and HLA‐B*35:03 (Px) molecules.

We first confirmed that the selected HIV peptide for our study, NY9, was immunogenic in our cohort of HLA‐B*35:01^+^HIV^+^ individuals. We showed that CD8^+^ T cells were able to develop a polyfunctional response after stimulation with the NY9 peptide. We then showed that the NY9 peptide that carries a tyrosine residue at PΩ was less stable in complex with HLA‐B*35:03 (Px) than HLA‐B*35:01 (PY). This agrees with the current classification within the Px and PY groups and the peptide‐binding motifs of these two allomorphs. The minimal information regarding HLA‐B*35:05 made it difficult to classify this molecule. Our data showed that HLA‐B*35:05 is stable while in complex with the NY9 peptide, suggesting that this allomorph belongs to the PY group. In addition, the crystal structures of the three allomorphs presenting the same HIV peptide showed that HLA‐B*35:01 and HLA‐B*35:05 can present the peptide in the same rigid conformation, while the NY9 peptide was highly mobile in the cleft of the HLA‐B*35:03 molecule. This further strengthens our hypothesis that the HLA‐B*35:05 molecule belongs to the PY group, because of its similar stability and ability to present peptide with a PΩ‐Tyr. This classification would align with the studies showing a lower viral load observed in HLA‐B*35:05^+^HIV^+^ patients than in HLA‐B*35:01^+^HIV^+^ patients and that HLA‐B*35:05 is a protective allele.^20^

We also observed some differences in the cleft of HLA‐B*35:05 compared with HLA‐B*35:01, namely, the loss of a hydrogen bond between residue 97 and the P9‐Tyr of NY9 peptide because of the substitution of Ser97 to Arg97. The smaller residues present within the cleft in the HLA‐B*35:05 molecule allowed for the binding of a small molecule. Indeed, we observed that the Bis‐Tris molecule was lodged in the HLA‐B*35:05 cleft underneath the NY9 peptide backbone. The Bis‐Tris molecule formed interactions with the P9‐Tyr of NY9 and the Ser97 of the HLA‐B*35:05, which would not be possible with an HLA carrying a large residue at position 97.

It was proposed that HLA‐B*35:05 had a reduced rigidity in the F pocket compared with HLA‐B*35:01 because of the amino acid substitution of residue 97, which is centrally positioned within the floor of the binding groove.^20^ Arginine is the most common residue at position 97 within the HLA‐B35 family, which confers risk to HIV‐1 disease.^36^ A smaller residue at position 97, as seen in HLA‐B*35:05, would disrupt the interactions of neighboring amino acids in the F pocket of the cleft, thereby altering the pocket flexibility. Pocket flexibility has been theorized to allow for higher self‐peptide binding during thymic selection, which may consequently decrease the T‐cell receptor repertoire that is responsive to HIV.^20^, ^37^ However, our data do not show higher flexibility of the F pocket in HLA‐B*35:05 compared with HLA‐B*35:01. The presence of the Bis‐Tris molecule in the cleft of the HLA‐B*35:05 molecule might stabilize the complex; however, in solution, the Bis‐Tris only moderately increases the HLA‐B*35:05 thermal stability assay. Therefore, it is possible that the F pocket of the HLA‐B*35:05 molecule is as stable as the one of the HLA‐B*35:01 molecule.

Comparison of the HLA‐B*35:03‐NY9 and HLA‐B*35:01‐NY9 structures revealed the different conformation the peptide adopts owing to the high mobility of its central part when bound to HLA‐B*35:03. Even though HLA‐B*35:01 and ‐B*35:03 only differ by one residue at the peptide‐binding site, this finding highlights the critical role of this residue at position 116 in the two groups (Px and PY) which seems to determine the preference for the PΩ of the peptide. However, the debate remains as to whether Px or PY is protective with the other being deleterious, as too few alleles have been classified into either subgroup. A recent study aimed to assess the Px subtype in the context of CD8^+^ T‐cell phenotypic profile with and without the presence of regulatory T cells. It was found that CD8^+^ T cells restricted by HLA‐B35‐Px exhibit an impaired phenotype that fails to elicit an efficient response to control viral replication and are thus directly associated with disease progression.^38^ As more HLA‐B35 molecules are classified into either group, with HLA‐B*35:05 now likely being a member of the PY group, this information might help to better understand the impact of this classification on disease and its progression.

Apart from classifying the previously unclassified HLA‐B*35:05 allele into the PY group, we also discovered that this molecule was able to bind a small molecule. Another instance where a small molecule binds to the antigen‐binding cleft was described for the HLA‐B*57:01, a protective allele against HIV progression.^39^ The binding of abacavir into the F pocket of HLA‐B*57:01 is leading to the activation of abacavir‐specific T cells and abacavir hypersensitivity syndrome in HIV^+^ patients.^32^, ^40^, ^41^

Interestingly, observations of drug sensitivity to the antiretroviral drug nevirapine in several populations support the association of hypersensitivity with specific HLA alleles with respect to ethnicity.^33^, ^34^, ^35^ A strong association of HLA‐B*35:05 allele expression and nevirapine‐induced skin rash was established in a population of HIV‐1^+^ Thai patients giving evidence for HLA‐B*35:05 as a predictor for hypersensitivity to the antiviral drug nevirapine.^33^ Furthermore, nevirapine‐induced cutaneous adverse drug reactions have been observed in HLA‐B35/HIV^+^ patients treated with the antiretroviral drug within an Indian population.^42^ As the HLA‐B*35:05‐NY9 crystal structure revealed the presence of a small molecule such as Bis‐Tris, it is possible that other small molecules such as drugs could bind in the cleft. As the Bis‐Tris molecule was contained in our crystallization buffer, this suggests that the small molecule binds once the peptide is already in the cleft, which is different from the previously described binding of abacavir to the HLA‐B*57:01 molecule.^32^ It remains to be seen whether other molecules, such as nevirapine, are able to bind into the HLA‐B*35:05 cleft.

## METHODS

### Sequence alignment

HLA‐B35 sequences were obtained from the IPD‐IMGT/HLA database.^25^ The residues within the peptide‐binding cleft of the HLA heavy chain (1–180) were used for the multiple sequence alignment, *via* the IPD‐IMGT/HLA Database Alignment Tool (https://www.ebi.ac.uk/ipd/imgt/hla/align.html; accessed on October 6, 2021).^25^ The amino acid differences and their positions within the HLA‐B35 sequence are summarized in Table 2.

### Protein expression, refold and purification

Protein expression, refold and purification were performed as described previously.^26^ In brief, DNA plasmids (GenScript, Hong Kong, China) encoding the HLA‐B*35:01, HLA‐B*35:03 and HLA‐B*35:05 heavy chain (1–275 amino acid) and β2‐microglobulin were transformed into the *BL21* strain of *Escherichia coli* cells. Both proteins were expressed separately as inclusion bodies and purified from the transformed *E. coli* cells. Soluble pHLA complexes were produced by refolding inclusion bodies in the following amounts: 30 mg of α‐chain, 10 mg of β2‐microglobulin and 5 mg of peptide (GenScript) into 200 mL of buffer (100 mm Tris–HCl pH 8.0, 400 mm l‐arginine, 500 μm glutathione oxidized, 5 mm glutathione reduced and 20 mm EDTA [ethylenediaminetetraacetic acid]) (Sigma Aldrich, Sydney, Australia). The refold mixture was dialyzed into 10 mm Tris–HCl pH 8.0 and the pHLA complexes were purified using anion exchange chromatography (HiTrap Q, Cytiva, Marlborough, MA, USA).

### Tetramer staining

CD8^+^ T‐cell lines were tetramer stained for 1 h at room temperature. Cells were washed and surface stained with anti‐CD8‐PerCP‐Cy5.5 (BD Biosciences, Melbourne, Australia), anti‐CD4‐BV650 (BD Biosciences), anti‐CD14‐APCH7, anti‐CD19‐APCH7 and Live/Dead Fixable Near‐IR Dead Cell Stain (Life Technologies, Melbourne, Australia). Cells were either fixed with 1% paraformaldehyde and acquired on a CytoFLEX Flow Cytometer or were directly single‐cell sorted for clonal expansion. Plates were centrifuged and stored at −80°C until use.

### Generation of HIV‐specific CD8 ^+^ T‐cell clones

Peripheral blood mononuclear cells from antiretroviral therapy–naïve individuals infected with HIV‐1, from the Perth HIV cohort study,^22^, ^23^ were isolated by Ficoll density centrifugation as previously described,^22^, ^23^ and frozen until needed. To identify HIV‐specific CD8^+^ T cells, PBMCs were thawed and stained on ice for 30 min with HLA‐B*35:01‐NY9 tetramer complexes (Leiden University Medical Centre, Leiden, the Netherlands and NIH Tetramer Core Facility, Emory University) and CD8‐APC (BD Biosciences, Perth, Australia). The cells were washed once in flow wash buffer (phosphate‐buffered saline with 1% heat‐inactivated fetal calf serum) and resuspended in 200 μL of flow wash buffer for flow cytometric analysis on the FACSCanto II.

HIV‐specific CD8^+^ T cells were then isolated by single‐cell sorting on the FACSAria III using the HLA‐B*35:01‐NY9 tetramer complex and either a combination of CD4‐FITC/CD19‐FTIC/CD45Ra‐FITC or CD8‐APC (BD Biosciences, Perth, Australia). The cells were sorted in 96‐well round‐bottom plates containing irradiated (30 Gy) allogeneic PBMCs, 120 IU interleukin‐2 (PeproTech, Rocky Hill, NJ, USA) and 80 ng/mL phytohemagglutinin (Sigma Aldrich) in a total volume of 100 μL of RPMI (Roswell Park Memorial Institute) 1640 (Life Technologies, Perth, Australia) supplemented with 5% heat‐inactivated fetal calf serum (Gibco, Thermo Fisher, Scoresby, Australia) and 5% heat‐inactivated human AB serum (Sigma Aldrich) and cultured at 37°C in an atmosphere of 5% CO_2_. On day 7 of the culture period, 100 μL of culture media (RPMI 1640 with 5% heat‐inactivated fetal calf serum and 5% human AB sera) and 100 IU interleukin‐2 were added to the wells, and the plates monitored for the outgrowth of clones.

### Confirmation of T‐cell clonality

To confirm the clonality of the HIV‐specific T‐cell clones, expression of the Vβ chain of the T‐cell receptors was initially determined using the TCR Vβ staining kit (Beckman Coulter, Brea, CA, USA). Individual tetramer‐specific CD8^+^ T‐cell clones expressing different Vβ chains were selected for further analysis and frozen until required. T‐cell receptor sequences were amplified using primer sequences as preciously described^43^ and sequencing of α‐chain (V and J) and β‐chain (V, D and J) CDR3 sequences was performed using next‐generation sequencing (Illumina MiSeq, San Diego, CA, USA) at the Institute for Immunology and Infectious Diseases (Murdoch University, Perth, Australia). Sequencing analysis was performed using the VDJFasta tool.^44^

### Intracellular cytokine staining assay

HLA‐B3501‐NY9‐specific CD8^+^ T‐cell clones were stimulated with 2 or 20 μm of NY9 peptide and were incubated for 7 h in the presence of GolgiPlug (BD Biosciences), GolgiStop (BD Biosciences) and anti‐CD107a‐AF488‐FITC (BD Biosciences). Following stimulation, cells were surface stained for 30 min with anti‐CD8‐PerCP‐Cy5.5 (BD Biosciences), anti‐CD4‐BV650 (BD Biosciences) and Live/Dead Fixable Near‐IR Dead Cell Stain (Life Technologies). Cells were fixed and permeabilized using BD Cytofix/Cytoperm solution (BD Biosciences) and then intracellularly stained with anti‐IFN‐γ‐BV421 (BD Biosciences) as well as anti‐TNF‐PeCy7, anti‐MIP1β‐APC and interleukin‐2‐PE (all BD Biosciences) for a further 30 min. Cells were acquired on a CytoFLEX Flow Cytometer. Post‐acquisition analysis was performed using FlowJo software (version 10.7.1; BD Biosciences). Cytokine detection levels identified in the no‐peptide control condition were subtracted from the corresponding test conditions in all summary graphs to account for nonspecific, spontaneous cytokine production.

### Thermal shift assay

To assess the stability of each HLA‐B35‐NY9 complex, a thermal‐shift (or thermal denaturation) assay was performed. The fluorescent dye SYPRO Orange (Invitrogen, Carlsbad, CA, USA) was used to monitor the protein unfolding. Real‐time fluorescence detection was conducted using Rotor‐Gene Q (QIAGEN, Hilden, Germany). Each HLA‐B*35‐NY9 complex was in 10 mm Tris–HCl pH 8.0 and 150 mm NaCl, at two concentrations (0.8 and 1.6 μm) in duplicate, and was heated from 25 to 95°C at a rate of 0.5°C min^−1^. Proteins subjected to Bis‐Tris or drug treatment were co‐incubated in 50 mm of Bis‐Tris or 2 μm of nevirapine (Sigma‐Aldrich, St Louis, MO, USA) for 1 h at 25°C before the thermal stability experiment. The fluorescence intensity was measured with excitation at 530 nm and emission at 557 nm. The data collected were processed and presented using GraphPad Prism 9 (version 9.2.0; GraphPad, San Diego, CA, USA). A sigmoidal nonlinear regression was performed. From the generated curve, the T_m_ (thermal melting temperature) was calculated, which represents the midpoint in the unfolding process (the temperature where 50% of the protein is unfolded).

### Crystallization and structure determination

Crystals of the pHLA complexes were grown *via* sitting drop vapor diffusion at 20°C with a protein‐to‐reservoir drop ratio of 1:1, at a concentration of 5, 4 and 2 mg mL^−1^ in 10 mm Tris–HCl pH 8.0, 150 mm NaCl for HLA‐B*35:01, HLA‐B*35:03 and HLA‐B*35:05, respectively. Crystals of HLA‐B*35:01 in complex with NY9 were grown in 15–25% polyethylene glycol (PEG) 4000, 0.2 m NH4 acetate and 0.1 m Na citrate pH 5.6 (Sigma Aldrich, Melbourne, Australia). pHLA crystals were soaked in a cryoprotectant solution containing mother liquor solution, with the PEG 4000 concentration increased to 30% (w/v) and then flash‐frozen in liquid nitrogen. Crystals of HLA‐B*35:03 in complex with NY9 were grown in 20% PEG 8000 and 0.1 m HEPES [4‐(2‐hydroxyethyl)‐1‐piperazineethanesulfonic acid] pH 7.5. pHLA crystals were soaked in a cryoprotectant solution containing mother liquor solution with the PEG 8000 concentration increased to 30% (w/v) and then flash‐frozen in liquid nitrogen. Crystals of HLA‐B*35:05 in complex with NY9 were grown in 25% PEG 3350 and 0.1 m Bis‐Tris pH 6.5. pHLA crystals were soaked in a cryoprotectant solution containing mother liquor solution with the PEG 3350 concentration increased to 30% (w/v) and then flash‐frozen in liquid nitrogen.

The data were collected on the MX2 beamline at the Australian Synchrotron, part of ANSTO, Australia.^45^ The data were processed using XDS^46^ and the structures were determined by molecular replacement using the PHASER program^47^ from the CCP4 suite^48^ with a model of HLA‐B*35:01 without the peptide (3LKO^49^). Manual model building was conducted using COOT^50^ followed by refinement with BUSTER.^51^ The final models have been validated and deposited using the wwPDB OneDep System (Supplementary Validation Reports [Link], [Link], [Link]) and the final refinement statistics and PDB codes are summarized in Table 4. All molecular graphics representations were created using the PyMOL molecular graphics system (version 2.4.2; copyright Schrödinger, LLC).

### Limitations of the study

Our study focuses on the HIV‐I epitope NY9 and certain HLA‐B35 alleles that have been reported to have preferences for particular amino acids at the F pocket. The NY9 has a tyrosine at position 9 (P9), which is preferred by HLA‐B35‐PY molecules indicated by high stability. It is possible that Px molecules may stably form complexes with other HIV‐I epitopes that have nontyrosine residues at P9.

## AUTHOR CONTRIBUTIONS

**Christian A Lobos:** Data curation; formal analysis; investigation; methodology; visualization; writing – original draft. **Demetra SM Chatzileontiadou:** Data curation; formal analysis; investigation; methodology; supervision; validation; visualization; writing – original draft; writing – review and editing. **Bonin Sok:** Data curation; formal analysis; investigation; methodology. **Coral‐Ann Almedia:** Investigation; methodology; writing – review and editing. **Hanim Halim:** Investigation; methodology; supervision; writing – review and editing. **Lloyd D'Orsogna:** Funding acquisition; investigation; methodology; project administration; supervision; writing – review and editing. **Stephanie Gras:** Conceptualization; formal analysis; funding acquisition; investigation; project administration; supervision; validation; writing – original draft; writing – review and editing.

## CONFLICT OF INTEREST

The authors declare no competing interests.

## RESOURCE AVAILABILITY

Further information and requests for resources and materials should be directed to the Lead Contact, Professor Stephanie Gras (s.gras@latrobe.edu.au).

## CODE AVAILABILITY STATEMENT

The final crystal structure models for the HLA‐B35‐NY9 complexes have been deposited to the Protein Data Bank (PDB) under the following accession codes: HLA‐B*35:01‐NY9 (7SIG), HLA‐B*35:03‐NY9 (7SIH) and HLA‐B*35:05‐NY9 (7SIF).

## Supporting information


Supplementary Validation Report 1

Supplementary Validation Report 2

Supplementary Validation Report 3

Supplementary figure 1

## Data Availability

All data that support the findings of this study are available in the Supporting Information of this article.
