# Supplementary material for: Molecular insights into the HLA‐B35 molecules' classification associated with HIV control
Source: Immunol Cell Biol. 2023 Oct 9;102(1):34–45. doi: 10.1111/imcb.12698 (PMC10952751; doi:10.1111/imcb.12698)
Supplement: Supplementary file 4 — Supplementary figure 1 [file IMCB-102-34-s003.docx]

**Supplementary figure 1. Gating strategy for our study, Related to Figure 1.**

HLA-B*35:01-NY9-specific CD8^+^ T cells clones gating strategy. CD8^+^ T cell responses were assessed by either tetramer staining or function in an ICS assay. Representative gating for the identification of CD8^+^ T cells in all assays (top panel). Representative gating for the assessment of polyfunctionality (middle panel). Representative flow cytometry plot of the identification of tetramer^+^ populations (bottom panel).

**
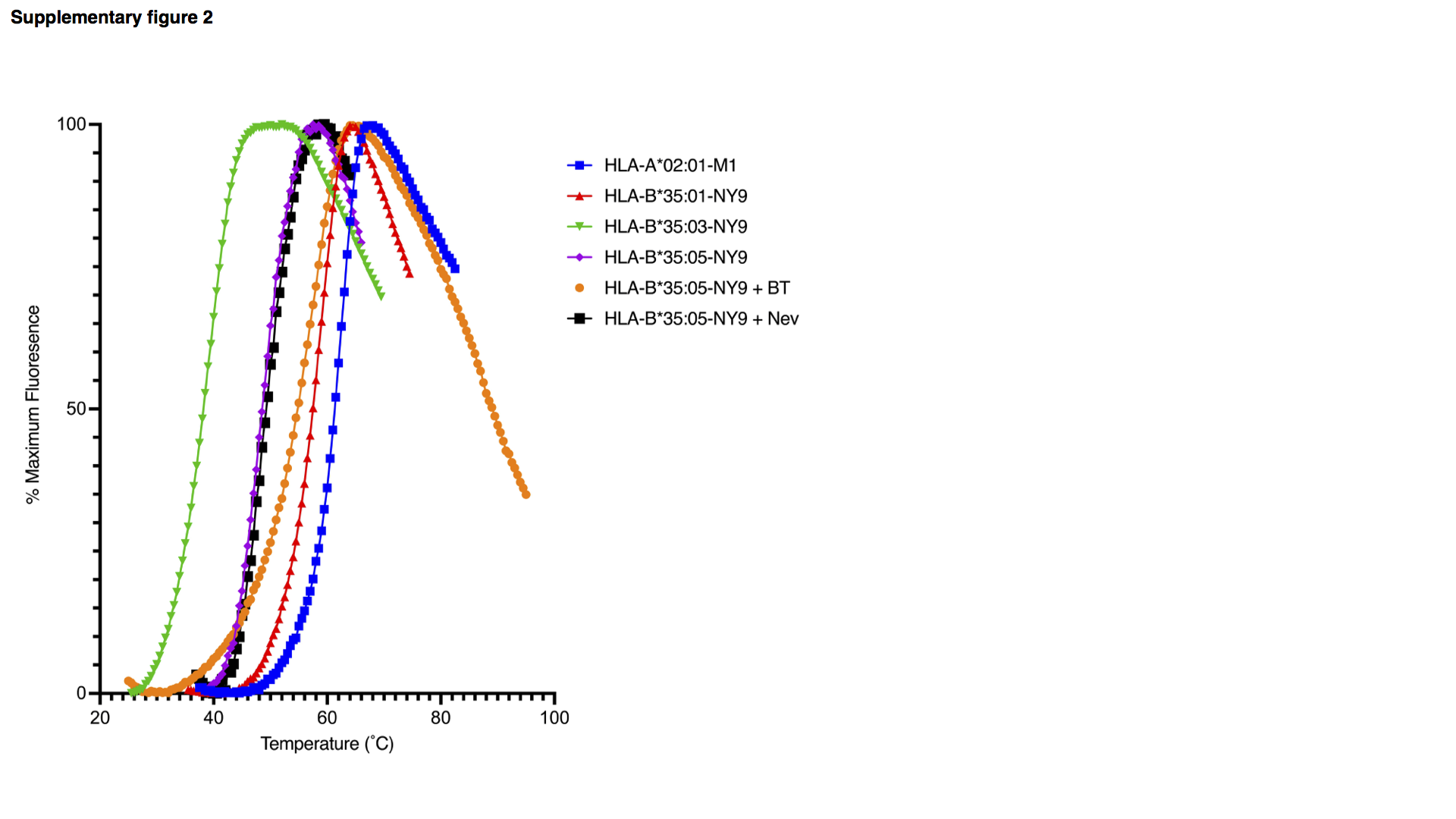
**

**Supplementary figure 2. Thermal denaturation.**

DSF plots showing normalized fluorescence intensity versus temperature for HLA-A*02:01-M1 (blue), HLA-B*35:01-NY9 (red), HLA-B*35:03-NY9 (green), HLA-B*35:05-NY9 (purple), HLA-B*35:05-NY9 with Bis-Tris (BT) (orange), and HLA-B*35:05-NY9 with Nevirapine (Nev) (black), measured at 0.8 and 1.6 μM concentrations (n=2). HLA-A*02:01-M1 complex was used as a control.

**
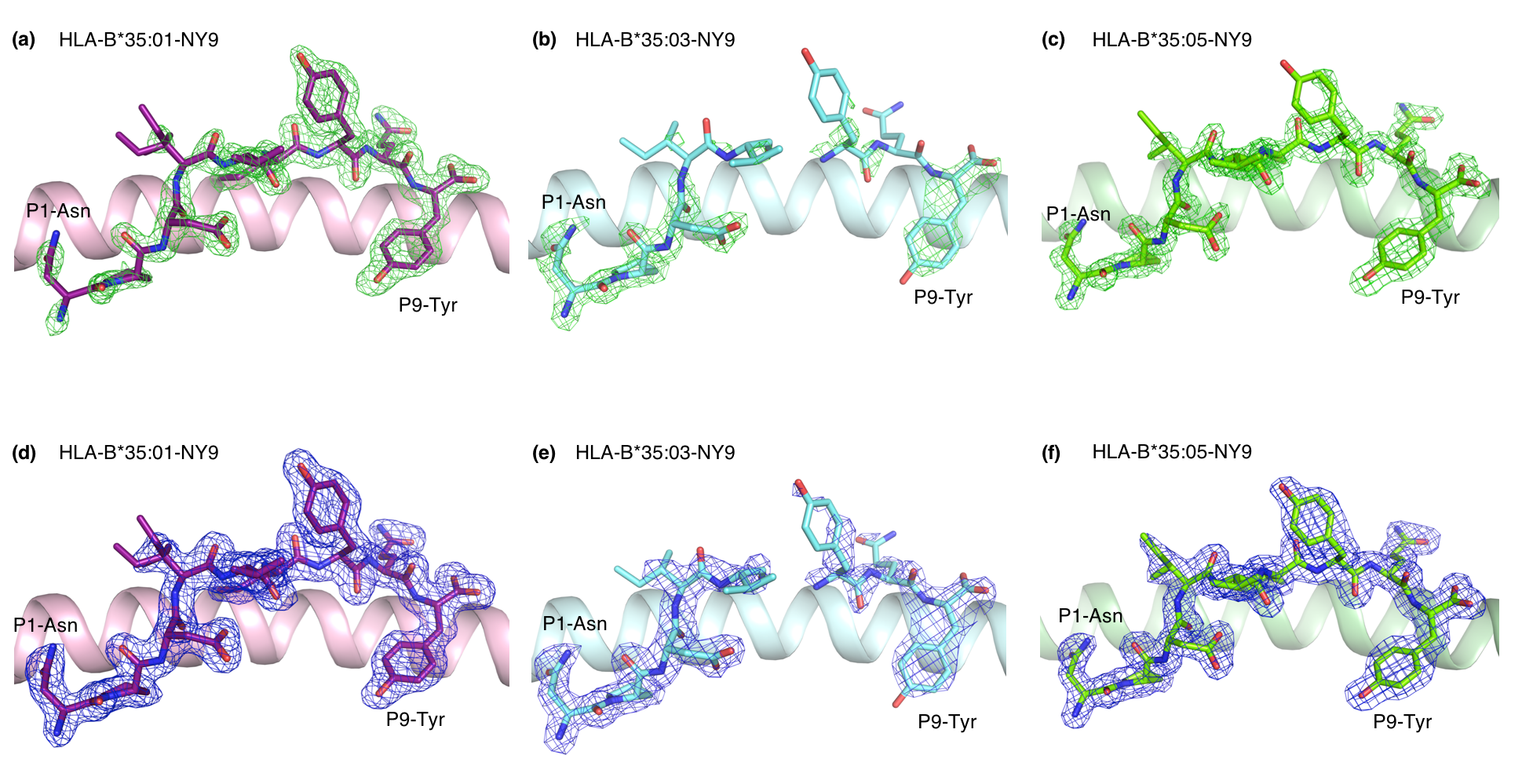
**

**Supplementary figure 3. Electron density maps of the NY9 peptide presented by HLA-B*35:01, -B*35:03, and -B*35:05 molecules, Related to Figure 2.**

HLA-B35 molecules are represented as cartoon, the NY9 peptide is represented as sticks. The **a-c** panels show the 2mFo-Fc electron density map (without peptide) contoured at 3σ and coloured in green, while **d-f** panels show the Fo-Fc electron density maps (with peptide) contoured at 1σ and coloured in blue.
